# Epithelial Protein Lost in Neoplasm α (Eplin-α) is transcriptionally regulated by G-actin and MAL/MRTF coactivators

**DOI:** 10.1186/1476-4598-9-60

**Published:** 2010-03-17

**Authors:** Laura Leitner, Dmitry Shaposhnikov, Arnaud Descot, Reinhard Hoffmann, Guido Posern

**Affiliations:** 1 AG Regulation of Gene Expression, Department of Molecular Biology, Max-Planck-Institute of Biochemistry, D-82152 Martinsried, Germany; 2 Institute of Medical Microbiology and Immunology, Technical University of Munich, D-81675 München, Germany

## Abstract

Epithelial Protein Lost in Neoplasm α is a novel cytoskeleton-associated tumor suppressor whose expression inversely correlates with cell growth, motility, invasion and cancer mortality. Here we show that Eplin-α transcription is regulated by actin-MAL-SRF signalling. Upon signal induction, the coactivator MAL/MRTF is released from a repressive complex with monomeric actin, binds the transcription factor SRF and activates target gene expression. In a transcriptome analysis with a combination of actin binding drugs which specifically and differentially interfere with the actin-MAL complex (Descot et al., 2009), we identified Eplin to be primarily controlled by monomeric actin. Further analysis revealed that induction of the Eplin-α mRNA and its promoter was sensitive to drugs and mutant actins which stabilise the repressive actin-MAL complex. In contrast, the Eplin-β isoform remained unaffected. Knockdown of MRTFs or dominant negative MAL which inhibits SRF-mediated transcription impaired Eplin-α expression. Conversely, constitutively active mutant actins and MAL induced Eplin-α. MAL and SRF were bound to a consensus SRF binding site of the Eplin-α promoter; the recruitment of MAL to this region was enhanced severalfold upon induction. The tumor suppressor Eplin-α is thus a novel cytoskeletal target gene transcriptionally regulated by the actin-MAL-SRF pathway, which supports a role in cancer biology.

## Findings

Epithelial Protein Lost in Neoplasm (referred to as Eplin) is a novel tumor suppressor affecting cell growth, cytoskeletal organisation and motility [1,2]. Eplin crosslinks, bundles and stabilises F-actin filaments and stress fibers, which correlates with its ability to suppress anchorage-independent growth in transformed cells [3-5]. In epithelial cells, Eplin is required for formation of the F-actin adhesion belt by binding to the E-cadherin-catenin complex through α-catenin [6].

**Figure 1:**
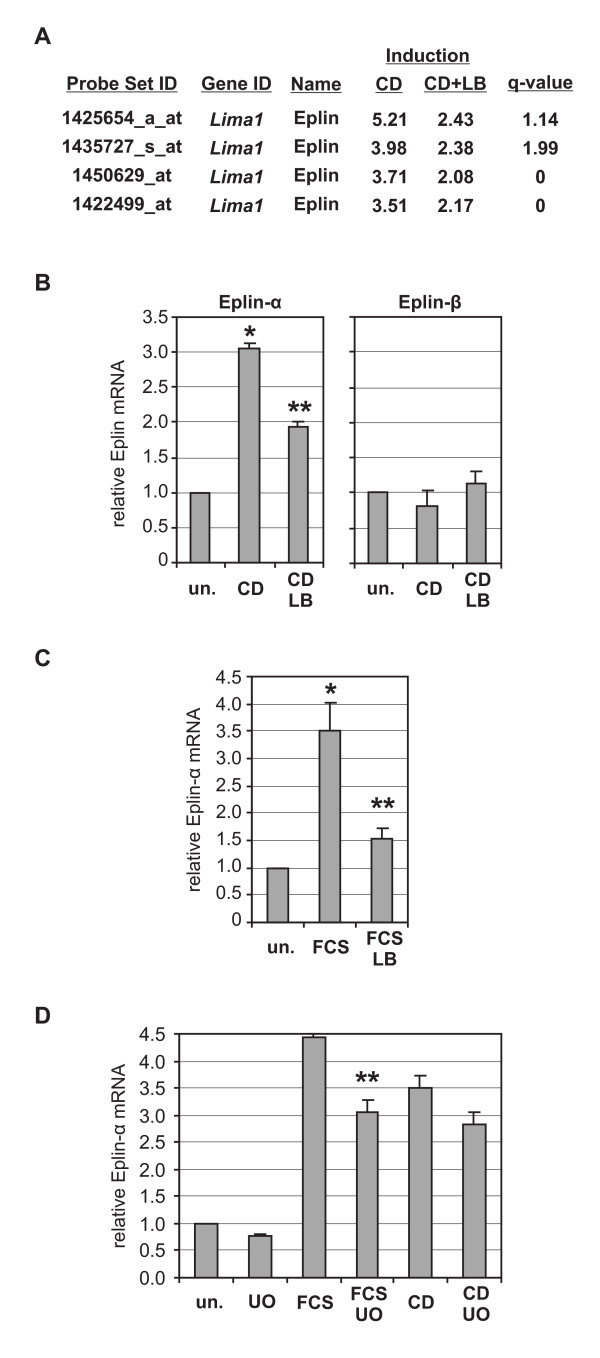
**Eplin-α expression is regulated by signalling through G-actin**. (A) Four independent Affymetrix probe sets of the *Lima1 *gene encoding Eplin were differentially regulated by actin binding drugs. G-actin regulated genes were induced by treatment with cytochalasin D (*CD*, 2 μM, 90 min) and repressed by latrunculin B (*LB*, 5 μM). Results shown are from transcriptome analysis of NIH 3T3 fibroblasts as previously described [19]. The q-value is the lowest false discovery rate at which the differentially expressed probe set is called significant. (B) Validation of differential regulation of Eplin-α, but not of Eplin-β, by actin binding drugs. NIH 3T3 cells were treated with cytochalasin D (2 μM) for 120 min, or with cytochalasin following 30 min pretreatment with latrunculin B (5 μM). Controls were left untreated (*un*.). The total mRNA was isolated and subjected to quantitative RT-PCR as described [19]. Shown is the average induction of Eplin mRNA after normalisation to *hprt*. *Error bars *indicate SEM (n = 3) for Eplin-α, and half range for Eplin-β. (C, D) Effect of pretreatment with latrunculin B (C) or UO126 (10 μM, 30 min) on the average induction of Eplin-α mRNA by serum (*FCS*, 15%, 90 min). *Error bars *indicate SEM of at least three independent experiments. The used primers were (positions of mRNA): Eplin-α, → (^1203^GCTGTTTCCGATGCTCCTAC^1223^), ← (^1382^CTCATTGTCGCTCTTGCT TG^1362^); Eplin-β, → (^183^CAAGAACAAGTCATCCGCAAT^204^), ← (^418^AGGAGGGTAGTCCGCTGTGT^398^). *Asterisk*, significant activation; *double asterisk*, significant repression (p < 0.01, unpaired student's t-test).

Eplin is encoded by *Lima1 *(LIM domain and actin binding-1) and expressed in two isoforms from distinct promoters: a longer Eplin-β (confusingly also called Eplin 1 or variant a) and a shorter Eplin-α (sometimes called Eplin 2 or variant b) [2,7]. Eplin-α mRNA is detected in various tissues and cell lines, but strikingly absent or downregulated in cancer cells [2]. In human breast cancer, its expression inversely correlates with poor prognosis, invasiveness and mortality [1]. Here we show that expression of the *Lima1 *gene is considerably affected by G-actin signalling (Fig. 1A).

Monomeric G-actin controls the activity of the transcription factor Serum Response Factor (SRF) by forming a repressive complex with its coactivator MAL/MRTF [8-10]. Upon Rho-family induced signal induction, MAL is released from actin, binds SRF and activates target gene expression [8,11-15]. Actin binding drugs differentially affect this subset of SRF target genes: treatment with cytochalasin D activates transcription by releasing MAL from G-actin, whilst latrunculin B stabilises the G-actin:MAL complex and inhibits gene expression [15-18].

Using this effect, we recently searched for G-actin regulated genes in NIH 3T3 cells by microarray expression analysis (GEO dataset GSE17105) [19]. Since both drugs depolymerised F-actin, genes depending on an intact cytoskeleton rather than on the G-actin switch did not score as differentially expressed. Strikingly, we found all four independent probe sets of the *Lima1 *gene (old Affymetrix annotation D15Ertd366e) to be differentially regulated (Fig. 1A). They were induced by cytochalasin between 3.5 and 5.2 fold, and simultanously repressed by latrunculin to 40-50%; statistical significance for differential regulation was high.

To validate our microarray result and to determine the regulated Eplin isoform, quantitative RT-PCR was performed. Endogenous Eplin-α mRNA was significantly upregulated by cytochalasin treatment, and this induction was repressed by latrunculin, whilst Eplin-β mRNA remained unaffected (Fig. 1B). This suggests that transcription from the Eplin-α promoter, but not from the Eplin-β promoter, is regulated by G-actin. In addition, Eplin-α was induced by serum independently from protein translation, consistent with its previous characterisation as an immediate-early serum responsive gene (Fig. 1C and data not shown) [7]. Importantly, the serum induction of Eplin-α was significantly inhibited by latrunculin pretreatment (Fig. 1C).

In parallel to Rho-actin signalling, serum stimulation also activates the MAPK pathway and facilitates SRF-dependent transcription through ternary complex factors (TCFs). To determine a potential role of MAPK signalling in Eplin-α transcription, cells were pretreated with the MEK inhibitor UO126. A slight but significant reduction of serum-induced Eplin-α mRNA was observed, whereas cytochalasin induction was essentially unaffected (Fig. 1D).

**Figure 2:**
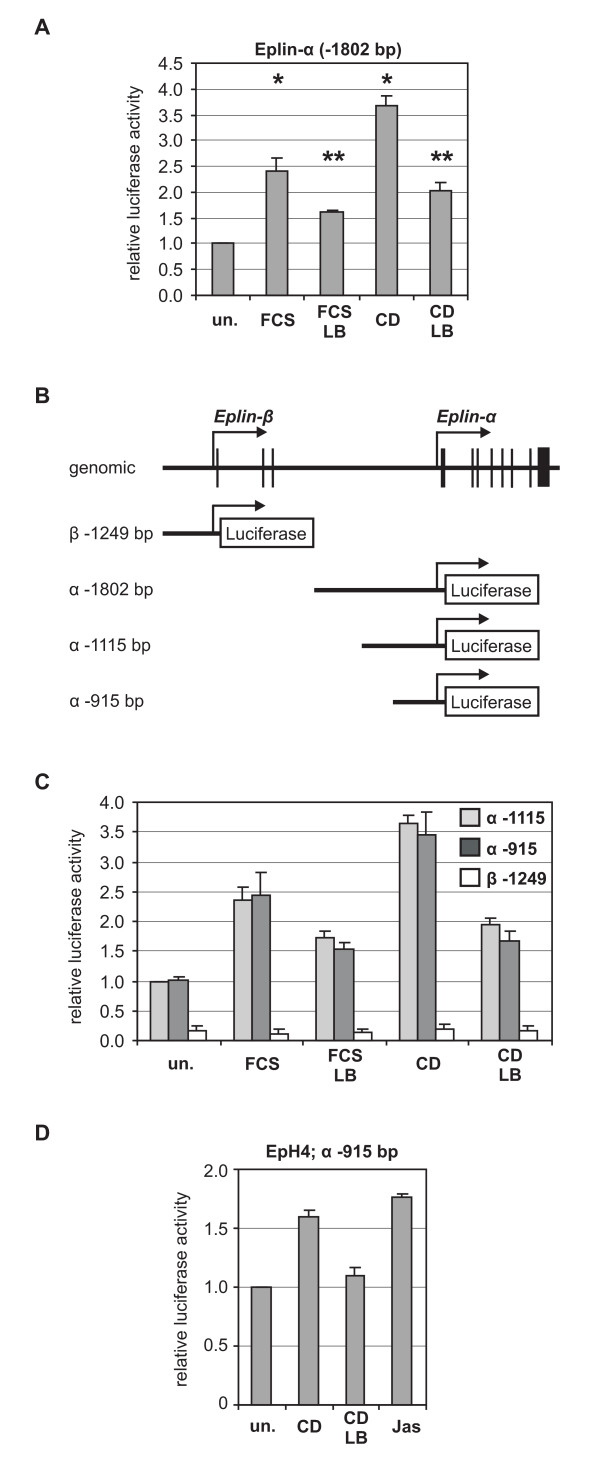
**The proximal promoter of Eplin-α is regulated through actin**. (A) The extended Eplin-α promoter confers regulation to a luciferase reporter gene. A genomic fragment covering nucleotides -1802 to +132 relative to the putative transcription start site of Eplin-α was cloned from murine liver tissue into the luciferase reporter plasmid pGL3. Following the indicated pretreatment with latrunculin B (5 μM, 30 min) and 7 hours of stimulation with serum (15%) or cytochalasin D (2 μM), transiently transfected NIH3T3 cells were lysed and the luciferase activity was determined as described [17]. (B) Schematic diagram of the genomic structure of the Eplin gene, and the promoter reporter constructs used. The Eplin-β reporter ranges from -1249 to +71, and the truncated Eplin-α fragments range from the indicated nucleotide to +284, relative to the transcription start site. (C) Analysis of the Eplin promoter reporter constructs by transient luciferase assays in NIH 3T3 fibroblasts. Shown is the mean relative luciferase activity, normalised to Renilla luciferase. *Error bars*, SEM (n = 3). (D) The proximal Eplin-α promoter is differentially regulated by actin binding drugs in mouse mammary epithelial EpH4 cells. Cells were transiently transfected with the Eplin-α (-915) promoter reporter construct, treated with cytochalasin, latrunculin, or jasplakinolide (0.5 μM, 7 h), and analysed as described [20]. Shown is the mean relative luciferase activity, with *Error bars *indicating half range. *Asterisk*, significant activation; *double asterisk*, significant repression (p < 0.05, unpaired student's t-test).

Next we investigated whether the Eplin-α promoter mediates regulation of heterologous luciferase reporter constructs, initially continuing with our NIH 3T3 model system. A 1934 bp fragment of the murine Eplin-α promoter, including the transcription start site, was activated by serum and cytochalasin, and its induction was reduced significantly by latrunculin pretreatment (Fig. 2A). To further identify a potential G-actin response element, several promoter deletions were analysed (Fig. 2B). In contrast to Eplin-β, all Eplin-α constructs were regulatable by serum, cytochalasin and latrunculin (Fig. 2C). This included the -915 construct, which lacks a possible SRF binding site around nucleotide -1050. The results suggest that a proximal promoter element is sufficient for Eplin-α regulation through actin. Consistent with this, the murine Eplin-α promoter harbours a consensus SRF binding site (CArG box) at -124 (gtCCTTATAAGGctatcctg), which is conserved in the human promoter; the Eplin-β promoter lacks any obvious CArG boxes [7].

Since Eplin-α was described as a protein enriched in epithelial cells, where it plays a critical role in adherens junction formation, we also determined the regulation in mouse mammary epithelial cells. Despite a generally lower level of induction by actin drugs in this cell type [20], the reporter was upregulated by both cytochalasin and jasplakinolide (an F-actin stabilising drug and inducer of actin-MAL signalling; [8]) and inhibited by latrunculin. This suggests that the mode of Eplin-α regulation is not fundamentally different in epithelial cells.

**Figure 3:**
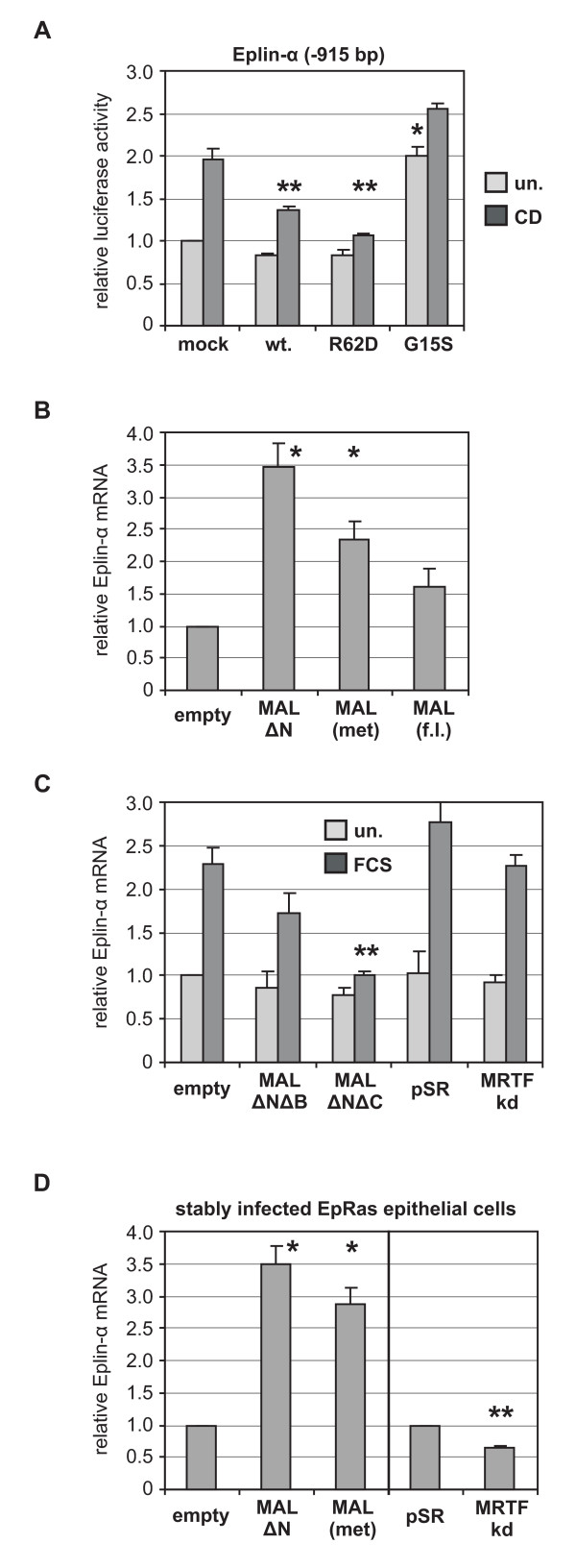
**Eplin-α expression is controlled by mutant actins and MAL/MRTF transcriptional coactivators**. (A) Cotransfection of NIH 3T3 cells with the indicated Eplin-α reporter and actin wildtype (*wt*), non-polymerisable mutant actin R62D, and F-actin stabilising mutant actin G15S [16,17]. Following cytochalasin treatment, the relative Luciferase activity was determined as before. (B) Transient expression of constitutively active MAL ΔN or MAL affect the Eplin-α mRNA level. Cells were transiently infected with the indicated pLPCX-derived retrovirus [19]. Two days later, the total mRNA was isolated and analysed for Eplin-α mRNA by quantitative RT-PCR as before. (C) Effect of dominant negative MAL ΔNΔB, MAL ΔNΔC, or double knockdown of MRTFs, on serum-stimulated Eplin-α expression. Infection was done with derivatives of either pLPCX or pSUPER-Retro (*pSR*, control), generating a shRNA targeting both MAL/MRTF-A and MRTF-B [19]. Two days after infection, cells were stimulated with serum if indicated (*FCS*, 15%, 90 min), and the relative Eplin-α mRNA induction was quantified. (D) Effect of constitutively active MAL ΔN and MAL, or double knockdown of MRTFs, on Eplin-α expression in EpRas epithelial cells. Shown is the average mRNA amount of EpRas cells stably infected with the indicated MAL constructs, compared to the mock-infected control cells (pLPCX and pSR control, respectively). *Error bars*, SEM (n = 3). *Asterisk*, significant activation; *double asterisk*, significant repression (p < 0.01, unpaired student's t-test).

A nonpolymerisable point mutant actin, R62D, is not incorporated into F-actin filaments, but increases the total cellular G-actin level, binds to MAL, and consequently inhibits SRF activation [8,17]. In contrast, G15S enhances F-actin formation and constitutively activates MAL and SRF independently from upstream signals [16]. Thus we ectopically expressed these actins together with the Eplin-α reporter to demonstrate its direct regulation by G-actin. Actin R62D as well as actin wildtype significantly inhibited the Eplin-α reporter induction by cytochalasin (Fig. 3A); a reduction was even observable following serum-stimulation (Additional file 1, Fig. S1A). Conversely, G15S activated Eplin-α in the absence of stimuli. Despite the low transfection rate of around 20%, similar effects were also observed on the total endogenous Eplin-α mRNA (Additional file 1, Fig. S1B). Moreover, it was previously shown that Eplin-α is activated by Rho family GTPases [7], which are critical inducers of G-actin:MAL dissociation [8]. This shows that the Eplin-α promoter resembles the regulation of MAL-dependent SRF target genes.

To directly test the effect of MAL on Eplin-α, we transiently infected cells with constitutively active MAL ΔN, which lacks the N-terminal actin-binding RPEL-motifs [8,18]. In addition, MAL(met) and MAL full length were analysed, which contain 2 or all 3 regulatory RPEL motifs, respectively. MAL ΔN and MAL(met) significantly induced endogenous Eplin-α mRNA expression (Fig. 3B). MAL full length exhibited the weakest activity, consistent with its tightest regulation through actin. This result demonstrated that MAL is sufficient to induce Eplin-α and identifies it as a MAL target gene.

Conversely, we determined whether MAL and MRTF-B are required for serum induction. Dominant negative constructs which either lack the SRF-binding basic region (ΔNΔB) or the C-terminal transactivation domain (ΔNΔC) were used. In addition, transient retroviral knockdown of both MAL and MRTF-B by shRNA was performed [19]. Interestingly, only MAL ΔNΔC significantly inhibited serum induction of Eplin-α, whereas ΔNΔB or a partial MRTF knockdown had little effect in NIH 3T3 cells (Fig. 3C). Consistent with this, MAL ΔNΔC is thought to block MAL/SRF function by forming a transcriptionally inactive SRF complex on target promoters. This complex, through interaction of the MAL basic region with the SRF B-box, does not permit simultanous formation of ternary complexes between SRF and the MAPK-regulated cofactors of the TCF-family [13,21]. In contrast, MAL ΔNΔB retains endogenous MAL via the leucine-zipper in an inactive, cytoplasmic state, but does not compete with TCF binding to the common SRF surface [13,22]. This suggests that Eplin-α can be regulated by both actin-MAL and MAPK-TCF signals. In line with this interpretation, serum induction of Eplin-α in fibroblasts is inhibited strongly by latrunculin and slightly by UO126 (Fig. 1C, D), and the Eplin-α promoter is known to contain TCF-binding sites adjacent to the SRF-binding CArG-box [7].

In order to determine the regulation of Eplin-α by MAL in epithelial cells, we stably infected mouse mammary epithelial cells with constitutively active MAL ΔN, MAL(met) and the MAL/MRTF-B knockdown construct. As observed in fibroblasts before, MAL ΔN and MAL(met) significantly induced endogenous Eplin-α mRNA expression in epithelial cells. (Fig. 3D). In addition, stable double knockdown of MRTFs decreased the endogenous Eplin-α mRNA level, showing that MRTFs are sufficient and required for Eplin-α expression in epithelial cells.

**Figure 4:**
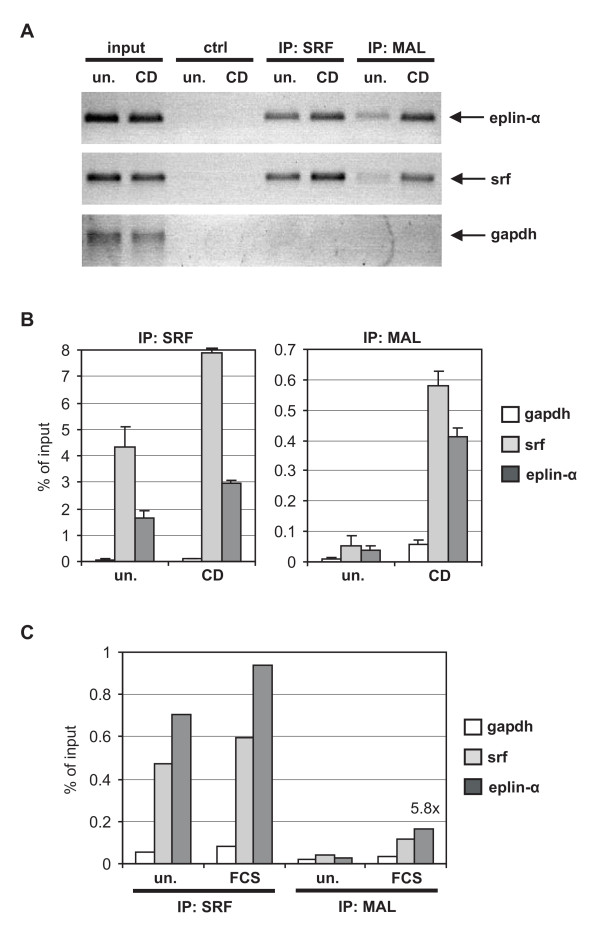
**Recruitment of MAL and SRF to the Eplin-α promoter**. Chromatin immunoprecipitation was performed using starved (*un*.) and stimulated NIH 3T3 cells (*CD*, 2 μM; *FCS*, 15%; 30 min), as indicated. Following chromatin preparation, antibodies specific for SRF (G-20, Santa Cruz) and MAL (homemade rabbit serum #79), or a negative control antibody (*ctrl*), were used for IP as described [19]. The primers used for amplifying an Eplin-α promoter fragment around the putative CarG box were: → (^-178^AAAAAGTCTCTCCCTTCCAATGT), ← (^-15^GTTACTGCCCTGCCACAAG). (A) Cytochalasin treatment induces recruitment of MAL to the Eplin-α and *srf *gene. Immunoprecipitated and input Eplin-α, *srf *and *gapdh *promoter fragments were amplified by conventional PCR and visualised by agarose gel electrophoresis. (B) Real-time PCR was performed from three independent chromatin preparations and IPs. Shown is the relative quantitation of *gapdh*, *srf *and Eplin-α promoter fragments in SRF and MAL immunoprecipitates, expressed in % of the input chromatin. *Error bars*, SEM (n = 3). (C) Quantitation of MAL and SRF recruitment to the Eplin-α, *srf *and *gapdh *gene following serum stimulation. Bound Eplin-α in MAL immunoprecipitates was increased 5.8 fold by FCS.

Finally, the recruitment of MAL and SRF to the Eplin-α promoter was analysed by chromatin immunoprecipitations from cells treated with and without cytochalasin. The known MAL-regulated *srf *gene and the *gapdh *gene were used as controls. Eplin-α was bound by SRF and inducibly recruited MAL upon cytochalasin stimulation, similar to the *srf *positive control (Fig. 4A). In contrast, neither the control antibody nor the *gapdh *gene resulted in a positive ChIP. Quantitive PCR following ChIP revealed that MAL recruitment to both Eplin-α and *srf *promoters is induced more than 10-fold, whilst SRF recruitment is only slightly enhanced (Fig. 4B). Moreover, SRF- and inducible MAL-recruitment were also detected after serum stimulation, comparable for *srf *and Eplin-α genes (Fig. 1C). Together, these results strongly suggest that the Eplin-α promoter is inducibly bound and regulated by MAL through its interaction with SRF.

Based on this study, we conclude that expression of the Eplin-α gene is transcriptionally regulated by actin-MAL signalling. The endogenous mRNA and the promoter of Eplin-α is induced by serum, actin binding drugs, Rho family members [7], constitutively active actin mutants, and MAL, and is negatively controlled by drugs which stabilise the repressive actin-MAL complex, by non-polymerisable actin mutants, and by dominant negative MAL or MRTF knockdown. In addition, the Eplin-α gene recruits SRF and, upon induction, MAL to a region containing a conserved CArG consensus site. The tumor suppressor Eplin-α is thus a novel cytoskeletal target gene regulated by the actin-MAL-SRF pathway. We note that high Eplin expression has been associated with stabilising less dynamic actin structures and enhanced adhesion, whereas SRF knockout and MRTF knockdown was shown to have the opposite effects [23,24]. Conversely, MAL overexpression results in enhanced cell spreading and antiproliferative effects [19]. Although MRTFs are required for experimental invasion and metastasis, the MAL homolog myocardin was recently described as a tumor suppressor [23,25]. Our study grants further investigations of the functional connections between MAL/MRTF family members and tumor suppressors such as Eplin-α during cancer progression.

## Abbreviations

CD: cytochalasin D; ChIP: chromatin immunoprecipitation; Eplin: Epithelial Protein Lost in Neoplasm; FCS: fetal calf serum; Hprt: hypoxanthine phosphoribosyltransferase; LB: latrunculin B; MAL/MRTF: Megakaryoblastic acute leukemia/myocardin related transcription factor; MAPK: mitogen-activated protein kinase; SRF: serum response factor; TCF: ternary complex factor.

## Competing interests

The authors declare that they have no competing interests.

## Authors' contributions

LL planned and performed the experiments and helped to draft the manuscript. DS carried out the chromatin IPs. AD and RH performed and analysed the microarrays. GP designed the study and drafted the manuscript. All authors read and approved the final manuscript.

## Supplementary Material

Additional file 1 **Actin mutants affect serum induction of Eplin-α**. (A) Cotransfection of NIH 3T3 cells with the indicated Eplin-α reporter and actin wildtype (*wt*), non-polymerisable mutant actin R62D, and F-actin stabilising mutant actin G15S [16,17]. One day later, cells were serum-starved (*un*., 0.5% FCS, 20 h) and stimulated (*FCS*, 15%, 7 h) if indicated, and the relative luciferase activity was determined as before. (B) Following transient transfection with the constructs indicated, cells were serum-starved for 40 h prior to serum-stimulation for 90 min as indicated. The total mRNA was isolated and analysed for Eplin-α mRNA by quantitative RT-PCR. Click here for file
