# Supplementary figures and images for: Epithelial Protein Lost in Neoplasm α (Eplin-α) is transcriptionally regulated by G-actin and MAL/MRTF coactivators
Source: Mol Cancer. 2010 Mar 17;9:60. doi: 10.1186/1476-4598-9-60 (PMC2848193; doi:10.1186/1476-4598-9-60)

**A**

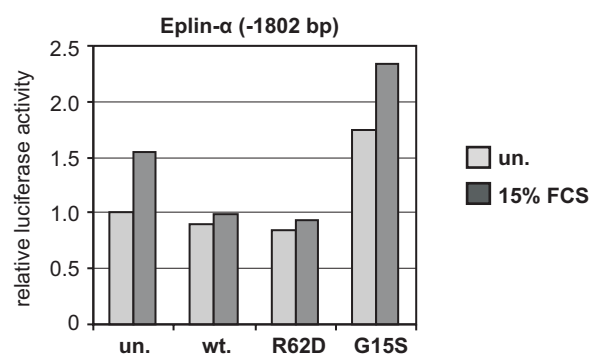

**B**

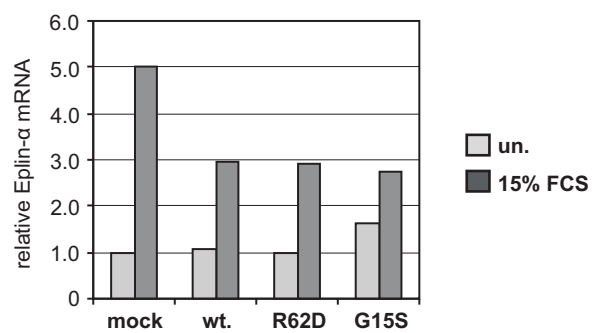

Supplement: Additional file 1 — Actin mutants affect serum induction of Eplin-α. (A) Cotransfection of NIH 3T3 cells with the indicated Eplin-α reporter and actin wildtype (wt), non-polymerisable mutant actin R62D, and F-actin stabilising mutant actin G15S [16,17]. One day later, cells were serum-starved (un., 0.5% FCS, 20 h) and stimulated (FCS, 15%, 7 h) if indicated, and the relative luciferase activity was determined as before. (B) Following transient transfection with the constructs indicated, cells were serum-starved for 40 h prior to serum-stimulation for 90 min as indicated. The total mRNA was isolated and analysed for Eplin-α mRNA by quantitative RT-PCR. [file 1476-4598-9-60-S1.PDF]
